# Racial Differences in Breast Cancer Treatment and Information Access

**DOI:** 10.1002/jso.70304

**Published:** 2026-07-05

**Authors:** Tokoya Williams, May X. Li, Keenan S. Fine, Bradley A. Melnick, Kelly C. Ho, Johna Joseph, Jayla Allums, Rolando J. Casas Fuentes, Brigid M. Coles, Robert D. Galiano

**Affiliations:** ^1^ Department of Surgery, Division of Plastic and Reconstructive Surgery Northwestern University Feinberg School of Medicine Chicago Illinois USA

**Keywords:** breast cancer, breast cancer treatment, breast reconstruction, racial disparities

## Abstract

**Background and Objectives:**

Persistent disparities in breast cancer (BC) care highlight the need to better understand patient experiences across diverse populations. This study examined racial differences in BC treatment, BR decisions, and social media use.

**Methods:**

A web survey of 413 racially diverse breast cancer survivors collected self reported clinical data, BREAST‐Q scores, and social media use. Multivariate regression examined BC treatment, BR complications, social media use, and satisfaction by race.

**Results:**

Black and Hispanic women were more likely to have mastectomy and chemotherapy than white women (*p* < 0.01; *p* < 0.001), even after adjusting for stage (*p* = 0.368). Asian women were less likely to receive chemotherapy or radiation (*p* < 0.01). Despite more breast‐conserving surgery (*p* < 0.001 vs. Black; *p* < 0.05 vs. Hispanic), white women reported lower breast satisfaction (*p* < 0.05). Black and Hispanic women relied more on social media for information (*p* < 0.001; *p* < 0.01). Younger age, tobacco use, radiation, and comorbidities increased BR complication risk.

**Conclusions:**

Women of color in this study experienced more aggressive treatment patterns and were more likely to rely on social media for information, highlighting an opportunity to address inequities in breast cancer care. Leveraging social media to deliver culturally tailored, evidence‐based information may enhance understanding of treatment and surgical options, as well as complication risks, thereby promoting more equitable, patient‐centered care.

## 1 Introduction

Women with breast cancer (BC) are increasingly choosing to undergo breast reconstruction (BR) after mastectomy [1]. BR has been demonstrated to enhance psychosocial outcomes and quality of life among breast cancer survivors [2, 3]. Despite these benefits, significant racial disparities in BR outcomes exist. White women who undergo BR surgery consistently report higher satisfaction, quality of decision, surgical access, and informational access compared to their Non‐white peers [4, 5, 6, 7]. Existing data largely focus on the experiences of caucasian women; thus, this study aims to fill in critical knowledge gaps in underrepresented Women of Color (WOC). Additionally, patients preparing to undergo BR have increasingly turned to social media to gather information, share experiences, and seek support [8, 9, 10]. However, recent studies have reported a lack of inclusion of dark‐skinned and non‐white individuals on plastic surgeons’ social media platforms [11]. Therefore, this study aimed to (1) investigate trends in BC treatment and decisions regarding BR surgery, and (2) assess the extent of social media engagement as a tool to support patients in their clinical decision‐making processes within a diverse population. Understanding these processes can help mitigate existing disparities in information access, healthcare access, breast cancer survival, and BR outcomes among women of color. Exploring these factors and how they relate to BC in a broader demographic can highlight disparities and contribute to a more equitable understanding of healthcare outcomes.

## 2 Materials and Methods

### 2.1 Participants and Survey Design

This study engaged a voluntary sample of 413 adult females with a history of surgical intervention for BC. A racially and ethnically diverse population was sampled via web‐based research platform Kantar Lightspeed LLC, including 250 white, 99 African American, 29 Hispanic, 24 Asian, and 11 American Indian/Alaska Native or Native Hawaiian or Other Pacific Islander (AI/AN or NH/PI) women. Race and ethnicity were combined into a single category based on survey platform design. Eligible participants were English‐speaking, cisgender women (18–75) with a self‐reported history of breast cancer; individuals outside this scope, including men and transgender respondents, were screened out. An online survey was developed in collaboration with the BRAVE Coalition that combined a 45‐item questionnaire, the 13‐item BREAST‐Q Satisfaction with Breasts subscale, and several open‐response questions [12]. The survey was developed with input from a multidisciplinary team, including a plastic surgeon, breast surgical oncologist, a PhD survey methodologist, a breast cancer survivor of color with research training, and a community advocate. It was administered from March 14, 2023, to June 30, 2024. Our target was at least 400 participants, with ≥ 50% identifying as women of color (respondents who self‐identified as non‐white), to ensure sufficient power for subgroup regression analyses while balancing cost and feasibility.

The 45‐item survey focused on self‐reported cancer stage and treatment to examine differences in chemotherapy, radiation, and surgical procedures across racial groups. Respondents were also asked about informational sources, including social media, and its role in breast cancer decision‐making. The survey included multiple‐choice, Likert‐style, and open‐ended questions to capture demographic and clinical variables, as well as decision‐making and perceptions of reconstruction. Branching logic was used to tailor questions based on responses, allowing participants such as those over age 65 or those undergoing breast‐conserving surgery to complete relevant sections without exclusion. To reduce survey fatigue while focusing on meaningful outcomes, we included the validated 13‐item BREAST‐Q Satisfaction with Breasts sub‐scale, which assesses physical and psychosocial aspects of postoperative satisfaction [13, 14, 15]. Kantar Lightspeed survey participants are typically compensated through a points‐based system, with rewards averaging $1 to $8 per survey, redeemable for cash, gift cards, or other incentives [16].

### 2.2 Recruitment and Sampling Methodology

Participants were recruited through Kantar Profiles (formerly Lightspeed), an open‐access survey platform with a vetted global panel. After study registration, eligible respondents were selected using Kantar's proprietary methods, which include AI‐driven fraud detection, stratified random sampling, and strict panel management to ensure data quality. The process emphasizes anonymity, unbiased framing, and random selection to minimize bias [13]. Eligible participants were English‐speaking adults (18–75) with a self‐reported history of breast cancer.

### 2.3 Statistical Analysis

Demographic and treatment‐related cancer data were analyzed using descriptive statistics. Continuous variables were expressed as mean ± standard deviation, while categorical data were summarized as frequencies and percentages. Associations between categorical variables were assessed using Chi‐squared tests, with Cramer's V employed to gauge the strength of those associations.

To explore factors influencing BR, univariate logistic regression analyzed predictors such as age at diagnosis and race/ethnicity. A multivariate logistic regression model was subsequently employed to determine independent predictors, with adjusted odds ratios (ORs) and 95% confidence intervals (CIs) reported. Associations between social media use and Breast‐Q scores were assessed using the Mann–Whitney test. Statistical significance was defined as *p* < 0.05. Data analysis was performed using R software (version 4.4.3) and Prism GraphPad (version 10.3.1).

## 3 Results

### 3.1 Patient Cancer Characteristics

At diagnosis, 76.3% of respondents had stage 0–2 breast cancer, while 23.7% had stage 3–4 disease. A total of 183 patients (44.3%) were diagnosed with Her2+ BC and 54 (13.1%) with triple‐negative BC (TNBC). Regarding treatment, 63.7% underwent chemotherapy, 67.3% received radiation therapy, and 81.6% had unilateral or bilateral mastectomy; 18.4% underwent breast‐conserving surgery (BCS). Among mastectomy patients, 64.4% received post‐mastectomy breast reconstruction (PMBR), of whom 56.7% had implant‐based and 43.3% autologous procedures.

Univariate analysis showed no significant difference in cancer stage among race groups (*p* = 0.368). Pairwise comparisons revealed no significant differences in the incidence of Her2+ BC or TNBC between white and Black, Hispanic, or AI/AN or NH/PI women. However, Asian women were significantly less likely to be diagnosed with either subtype compared to white women (Table 1).

**TABLE 1:** Patient characteristics of all respondents.

Variable	All (*N* = 413)	White (*N* = 250)	Black (*N* = 99)	Hispanic (*N* = 29)	American Indian/Alaska Native (*N* = 11)	Asian (*N* = 24)	Univariate *p* value
Age Range							0.106
Less than 35	47 (21.3%)	22 (18.2%)	15 (25.4%)	8 (44.4%)	1 (14.3%)	1 (6.3%)	
35–44	50 (22.6%)	29 (24.0%)	10 (17.0%)	4 (22.2%)	2 (28.6%)	5 (31.3%)	
45–54	36 (16.3%)	21 (17.4%)	6 (10.2%)	2 (11.1%)	2 (28.6%)	5 (31.3%)	
55–64	58 (26.2%)	27 (22.3%)	22 (37.3%)	4 (22.2%)	2 (28.6%)	3 (18.8%)	
65 or over	30 (13.6%)	22 (18.2%)	6 (10.2%)	0 (0.0%)	0 (0.0%)	2 (12.5%)	
Average BMI (kg/cm2)	28.4 (8.5)a	28.4 (9.0)a	29.3 (8.4)a	28.8 (4.4)a	30.3 (12.2)ab	23.6 (3.5)b	0.006
Tumor Biology							0.025
Her2 positive	183 (44.3%)	102 (40.8%)a	51 (51.5%)a	17 (58.6%)a	5 (45.5%)ab	8 (33.3%)b	
Triple Negative	54 (13.1%)	31 (12.4%)a	16 (16.2%)a	5 (17.2%)a	2 (18.2%)ab	0 (0.0%)b	
Neither	86 (20.8%)	55 (22.0%)	15 (15.2%)	2 (6.9%)	3 (27.3%)	11 (45.8%)	
Unsure	90 (21.8%)	62 (24.8%)	17 (17.2%)	5 (17.2%)	1 (9.1%)	5 (20.8%)	
Cancer Stage							0.368
DCIS (Stage 0)	70 (16.9%)	46 (18.4%)a	17 (17.2%)b	2 (6.9%)bc	0 (0.0%)cd	5 (20.8%)abd	
Stage I	110 (26.6%)	59 (23.6%)a	34 (34.3%)b	6 (20.7%)bc	5 (45.5%)cd	6 (25.0%)abd	
Stage II	135 (32.7%)	86 (34.4%)a	24 (24.2%)b	11 (37.9%)bc	3 (27.3%)cd	11 (45.8%)abd	
Stage III	72 (17.4%)	40 (16.0%)a	19 (19.2%)b	8 (27.6%)bc	3 (27.3%)cd	2 (8.3%)abd	
Stage IV	10 (2.4%)	6 (2.4%)a	3 (3.0%)b	1 (3.4%)bc	0 (0.0%)cd	0 (0.0%)abd	
Unsure	16 (3.9%)	13 (5.2%)	2 (2.0%)	1 (3.4%)	0 (0.0%)	0 (0.0%)	
Breast Surgery							0.215
Mastectomy	337 (81.6%)	200 (80.0%)	85 (85.9%)	25 (86.2%)	9 (81.8%)	18 (75.0%)	
Breast‐Conserving Surgery or Oncoplastic Breast Surgery	76 (18.4%)	50 (20.0%)	14 (14.1%)	4 (13.8%)	2 (18.2%)	6 (25.0%)	
Radiation Therapy							0.123
Yes	278 (67.3%)	174 (69.6%)a	67 (67.7%)a	19 (65.5%)ab	9 (81.8%)a	9 (37.5%)b	
No	132 (32.0%)	74 (29.6%)a	31 (31.3%)a	10 (34.5%)ab	2 (18.2%)a	15 (62.5%)b	
Unsure	3 (0.7%)	2 (0.8%)	1 (1.0%)	0 (0.0%)	0 (0.0%)	0 (0.0%)	
Chemotherapy							0.002
Yes	263 (63.7%)	144 (57.6%)a	74 (74.7%)a	24 (82.8%)ab	8 (72.7%)ab	13 (54.2%)b	
No	145 (35.1%)	103 (41.2%)a	25 (25.3%)a	4 (13.8%)ab	2 (18.2%)ab	11 (45.8%)b	
Unsure	5 (1.2%)	3 (1.2%)	0 (0.0%)	1 (3.4%)	1 (9.1%)	0 (0.0%)	

a, b, c indicate statistically significant differences between groups based on the specified post hoc multiple comparisons test, such that values sharing the same letter are not significantly different from each other (*p* < 0.05).

### 3.2 Breast Cancer Treatment by Race

Concerning BC treatment, multivariate logistic regression found that patients in all age groups over 45 were less likely to undergo chemotherapy than patients < 35 years old (*p* < 0.05). Notably, Black women were significantly more likely to undergo chemotherapy 2.600 [1.322−5.272], *p* < 0.01) compared to non‐Black women, regardless of BC stage, BC type, age, comorbidities, income, and education level. Further regression analysis showed that Black and Hispanic women were significantly more likely to undergo mastectomy (5.350 [2.263−14.943], *p* < 0.001 and 5.582 [1.081−102.603], *p* < 0.05 respectively) after controlling for cancer stage, cancer type, age, comorbidities, income, and education level (Table 2).

**TABLE 2:** Multivariate logistic regression predictors of breast cancer treatment.

Predictor	Chemotherapy	Radiation	Mastectomy
Age Group			
< 35	1.0 [Reference]	1.0 [Reference]	1.0 [Reference]
35–45	0.282 [0.067−0.989]	0.712 [0.267−1.857]	0.497 [0.167−1.423]
45–54	0.150 [0.036−0.513]**	0.376 [0.142−0.954]*	0.263 [0.089−0.734]*
55–64	0.244 [0.063−0.749]**	0.548 [0.227−1.249]	0.396 [0.145−1.030]
65+	0.067 [0.017−0.208]***	0.485 [0.197−1.136]	0.276 [0.107−0.672]*
Race/Ethnicity			
White	1.0 [Reference]	1.0 [Reference]	1.0 [Reference]
Black	2.600 [1.322−5.272]**	0.866 [0.488−1.550]	5.350 [2.263−14.943]***
Hispanic	2.210 [0.631−9.302]	0.303 [0.109−0.816]	5.582 [1.081−102.603]*
AI/AN	1.803 [0.381−13.106]	1.596 [0.368−11.111]	2.650 [0.434−51.449]
Asian	1.306 [0.414− 24.233]	0.303 [0.109−0.816]*	2.924 [0.740−19.621]
Cancer Stage	3.813 [2.799−5.357]***	2.050 [1.609−2.648]***	2.160 [1.588−3.001]***

*Note:* Cancer Stage is treated as an ordinal variable, where the odds ratio (OR) represents the increased likelihood of receiving chemotherapy, radiation, and mastectomy with each progression in the cancer stage from stage 0 to stage IV.

*p < 0.05; **p < 0.01; ***p < 0.001.

**FIGURE 1:**
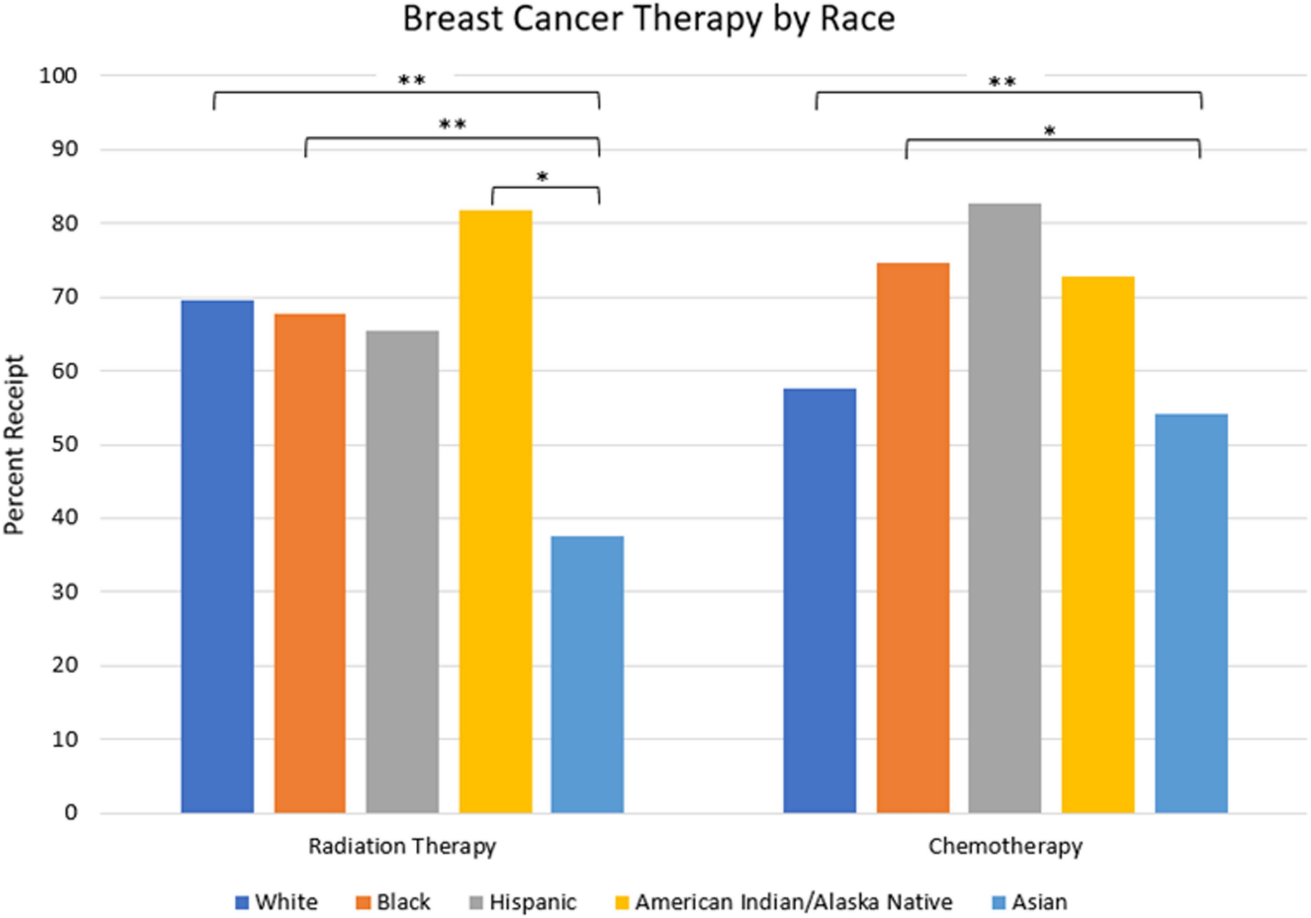
Univariate analysis demonstrated that Asian patients were significantly less likely to receive radiation therapy compared to White, Black, and AI/AN patients (*p = 0.010, **p = 0.003, *p = 0.038, respectively), and that Asian patients were significantly less likely to receive chemotherapy compared to White and Black patients (**p = 0.004, *p = 0.017, respectively). Total respondents = 413 (White = 250, Black = 99, Hispanic = 29, Asian = 24, AI/AN = 11). Asterisks indicate statistical significance: *p < 0.05, **p < 0.01.

Univariate analysis demonstrated no statistically significant differences in the rates of radiation therapy receipt between white, Black, Hispanic, and AI/AN patients. Pairwise comparison showed that Asian women were significantly less likely to undergo radiation therapy compared to their white (*p* = 0.003), Black (*p* = 0.010), and AI/AN (*p* = 0.038) patients. Asian women were also significantly less likely to undergo chemotherapy compared to white and Black women (*p* = 0.004 and *p* = 0.017, respectively) (Figure 1). Regression analysis showed that Asian women were significantly less likely to undergo radiation therapy, regardless of cancer stage, age, comorbidities, income, and education level (Table 2).

### 3.3 Breast Reconstruction Complications, Outcomes, and Comorbidities

**FIGURE 2:**
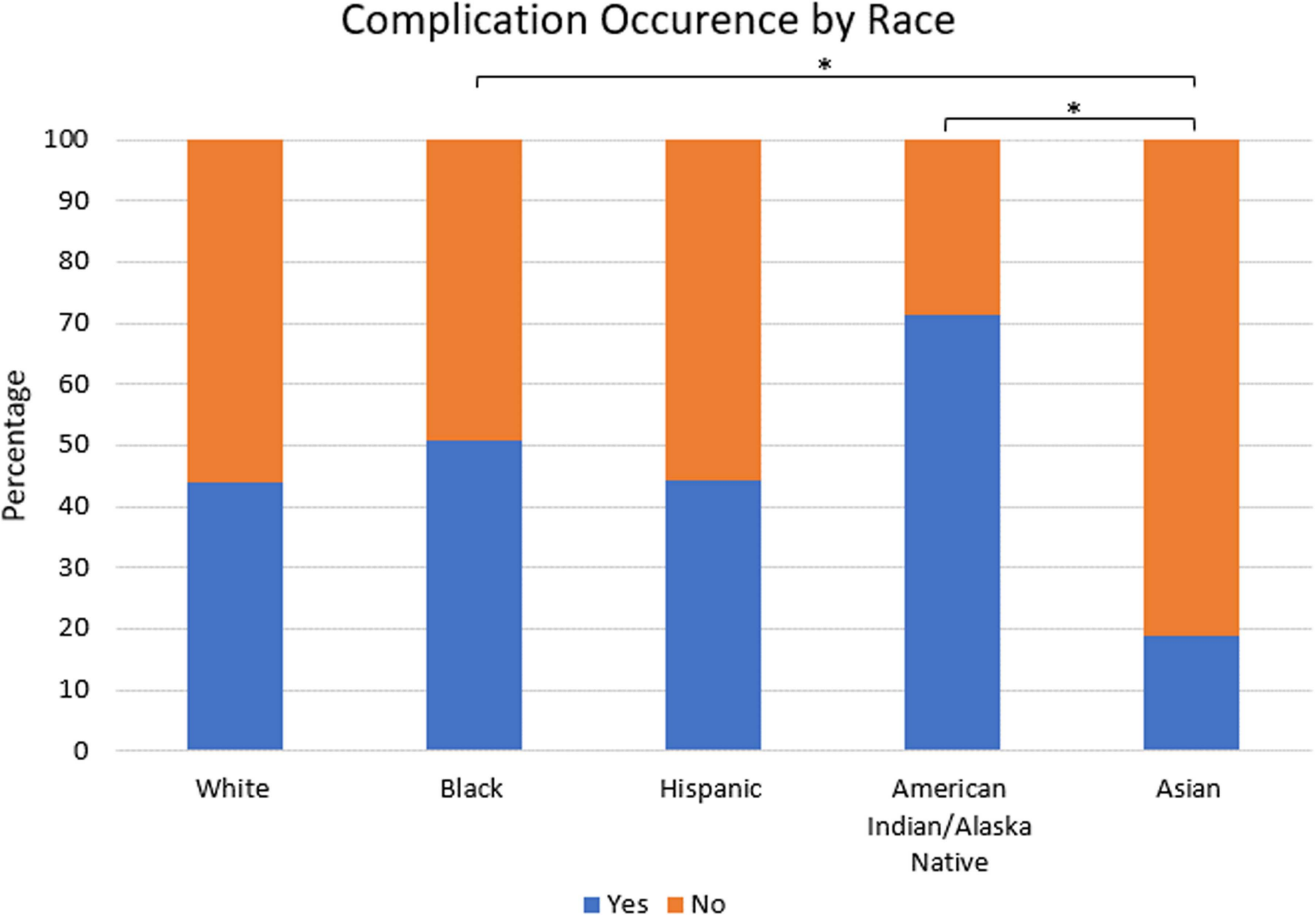
Univariate analysis demonstrated that Asian patients were significantly less likely to experience complications following breast reconstruction compared to Black and AI/AN patients (*p = 0.022, *p = 0.015, respectively). Total respondents = 413 (White = 250, Black = 99, Hispanic = 29, Asian = 24, AI/AN = 11). Asterisks indicate statistical significance: *p < 0.05.

Among patients who underwent BR surgery (Table 3), univariate analysis showed that younger age (*p* < 0.05), radiation therapy (*p* < 0.001), tobacco use (*p* < 0.001), diabetes (*p* < 0.001), heart disease (*p* < 0.01), and autologous BR (*p* < 0.05) each significantly increased postoperative complication risk (Table 4). Overall, 62.4% of patients reported at least one medical comorbidity: heart disease (8.0%), hypertension (45.5%), diabetes (19.6%), renal disease (5.8%), and dyslipidemia (31.0%). Black patients were more likely than white patients to report diabetes (*p* < 0.004), with a trend toward higher hypertension rates (*p* = 0.055). No significant differences were observed between white and Hispanic patients for diabetes (*p* = 0.07) or hypertension (*p* = 0.191). Regression analysis identified tobacco use (*p* < 0.001) and autologous BR (*p* < 0.05) as independent predictors of postoperative complications (Table 5). Asian patients were significantly less likely to experience complications compared to other race groups, though this finding is limited by small sample size (Figure 2). No other racial differences reached statistical significance.

**TABLE 3:** Patient characteristics of respondents who underwent breast reconstruction.

Variable	All (*N* = 221)	White (*N* = 121)	Black (*N* = 59)	Hispanic (*N* = 18)	American Indian/Alaska Native (*N* = 7)	Asian (*N* = 16)	Univariate *p* value
Breast Reconstruction Surgery							0.044
Implant‐based	123 (55.7%)	69 (57.0%)a	26 (44.1%)a	12 (66.7%)ab	2 (28.6%)a	14 (87.5%)b	
Autologous or flap	90 (40.7%)	46 (38.0%)ab	32 (54.2%)a	6 (33.3%)ab	4 (57.1%)a	2 (12.5%)b	
Unsure	4 (1.8%)	3 (2.5%)	1 (1.7%)	0 (0.0%)	0 (0.0%)	0 (0.0%)	
Other	4 (1.8%)	3 (2.5%)	0 (0.0%)	0 (0.0%)	1 (14.3%)	0 (0.0%)	
Any Complication							0.12
Yes	99 (44.8%)	53 (43.8%)ab	30 (50.8%)a	8 (44.4%)ab	5 (71.4%)a	3 (18.8%)b	
No	122 (55.2%)	68 (56.2%)ab	29 (49.2%)a	10 (55.6%)ab	2 (28.6%)a	13 (81.3%)b	
Type of Complication							
Seroma	22 (12.3%)	13 (13.3%)	4 (8.3%)	2 (12.5%)	2 (18.2%)	1 (16.7%)	
Hematoma	24 (13.4%)	16 (16.3%)	4 (8.3%)	1 (6.3%)	2 (18.2%)	1 (16.7%)	
Infection	35 (19.6%)	14 (14.3%)	11 (22.9%)	5 (31.3%)	2 (18.2%)	3 (50.0%)	
Wound dehiscence	26 (14.5%)	17 (17.3%)	4 (8.3%)	2 (12.5%)	2 (18.2%)	1 (16.7%)	
Flap compromise or loss	36 (20.1%)	13 (13.3%)	17 (35.4%)	4 (25.0%)	2 (18.2%)	0 (0.0%)	
Capsular Contracture	30 (16.8%)	22 (22.4%)	5 (10.4%)	2 (12.5%)	1 (9.1%)	0 (0.0%)	
Other	6 (3.4%)	3 (3.1%)	3 (6.3%)	0 (0.0%)	0 (0.0%)	0 (0.0%)	

a, b, c indicate statistically significant differences between groups based on the specified post hoc multiple comparisons test, such that values sharing the same letter are not significantly different from each other (*p* < 0.05).

**TABLE 4:** Univariate predictors of complications after breast reconstruction surgery.

	No Complication	Complication	*p* value
Age Group			
< 35	20 (42.6%)	27 (57.4%)	*p* = 0.02875*
35–44	25 (50.0%)	25 (50.0%)	
45–54	25 (69.4%)	11 (30.6%)	
55–64	30 (51.7%)	28 (48.3%)	
65+	22 (73.3%)	8 (26.7%)	
Race			
AI/AN	2 (28.6%)	5 (71.4%)	*p* = 0.120
Asian	13 (81.3%)	3 (18.8%)	
Black	29 (49.2%)	30 (50.8%)	
Hispanic	10 (55.6%)	8 (44.4%)	
White	68 (56.2%)	53 (43.8%)	
Radiation			
No	59 (70.2%)	25 (29.8%)	
Yes	63 (46.0%)	74 (54.0%)	*p* < 0.001***
Tobacco			
No	104 (69.8%)	45 (30.2%)	
Yes	18 (25.0%)	54 (75.0%)	*p* < 0.001***
BR Type			
Autologous	41 (45.6%)	49 (54.4%)	*p* = 0.027*
Implant	76 (61.8%)	47 (38.2%)	
Obesity (BMI > 30 kg/m^2^)			
No	80	51	*p* = 0.911
Yes	36	25	

*p < 0.05; ***p < 0.001.

**TABLE 5:** Tobacco use and autologous breast reconstruction are independent risk factors for BR complications.

Predictor	Odds Ratio	2.5% CI	97.5% CI	*p* value
Tobacco Use	8.1405	3.5366	20.0429	***p*** < 0.001***
Autologous Breast Reconstruction	2.2419	1.1316	4.5522	***p*** = 0.0224*

*p < 0.05; ***p < 0.001.

### 3.4 Information Access

**FIGURE 3:**
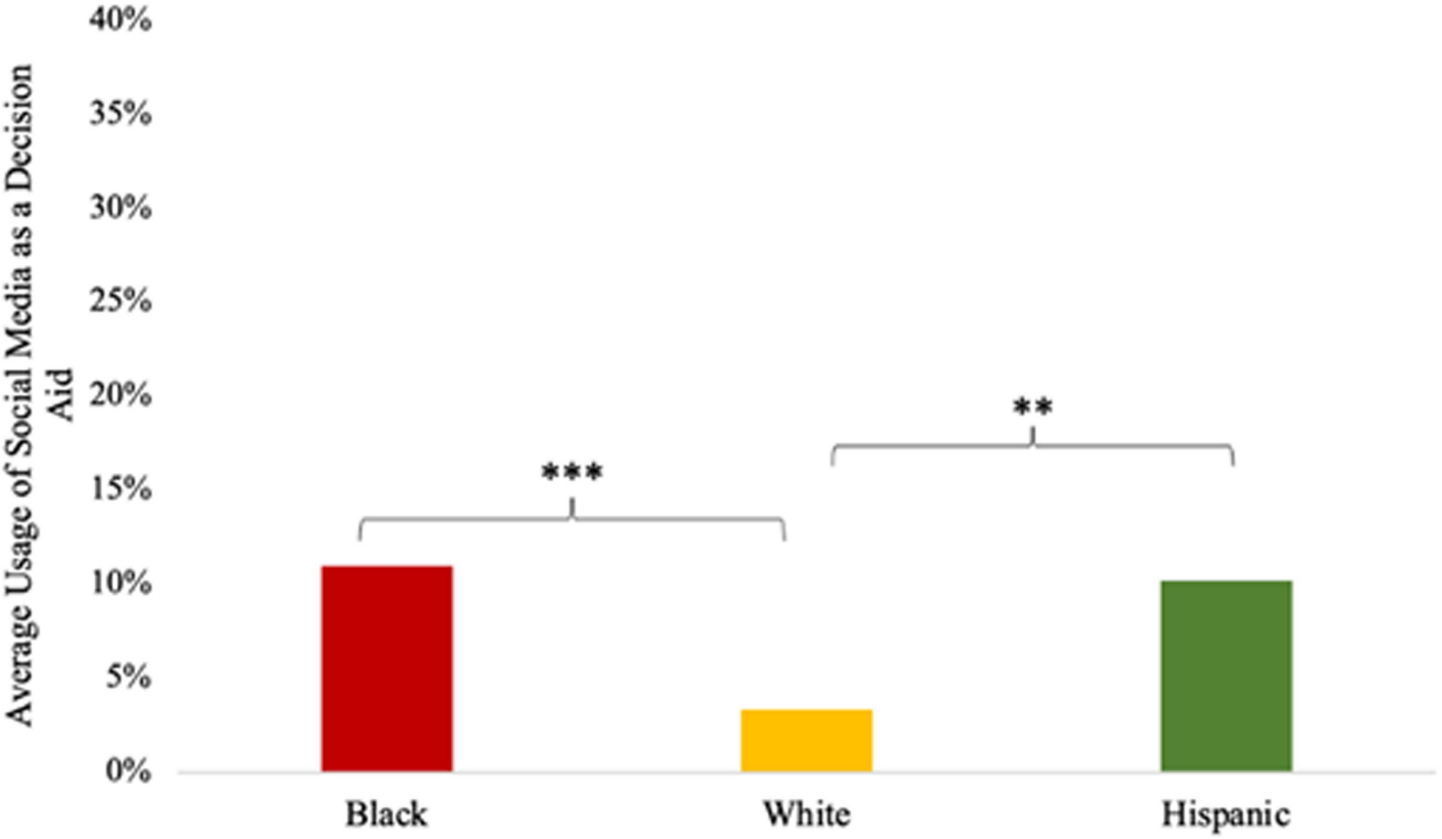
Social media as a decision aid for breast reconstruction. Black and Hispanic patients were significantly more likely to consult social media compared to White patients (**p < 0.01, ***p < 0.001, respectively). Total respondents = 378 (White = 250, Black = 99, Hispanic = 29). Asian and AI/AN groups were not included due to small sample sizes, which limited statistical power. Asterisks indicate statistical significance: **p < 0.01, ***p < 0.001.

**FIGURE 4:**
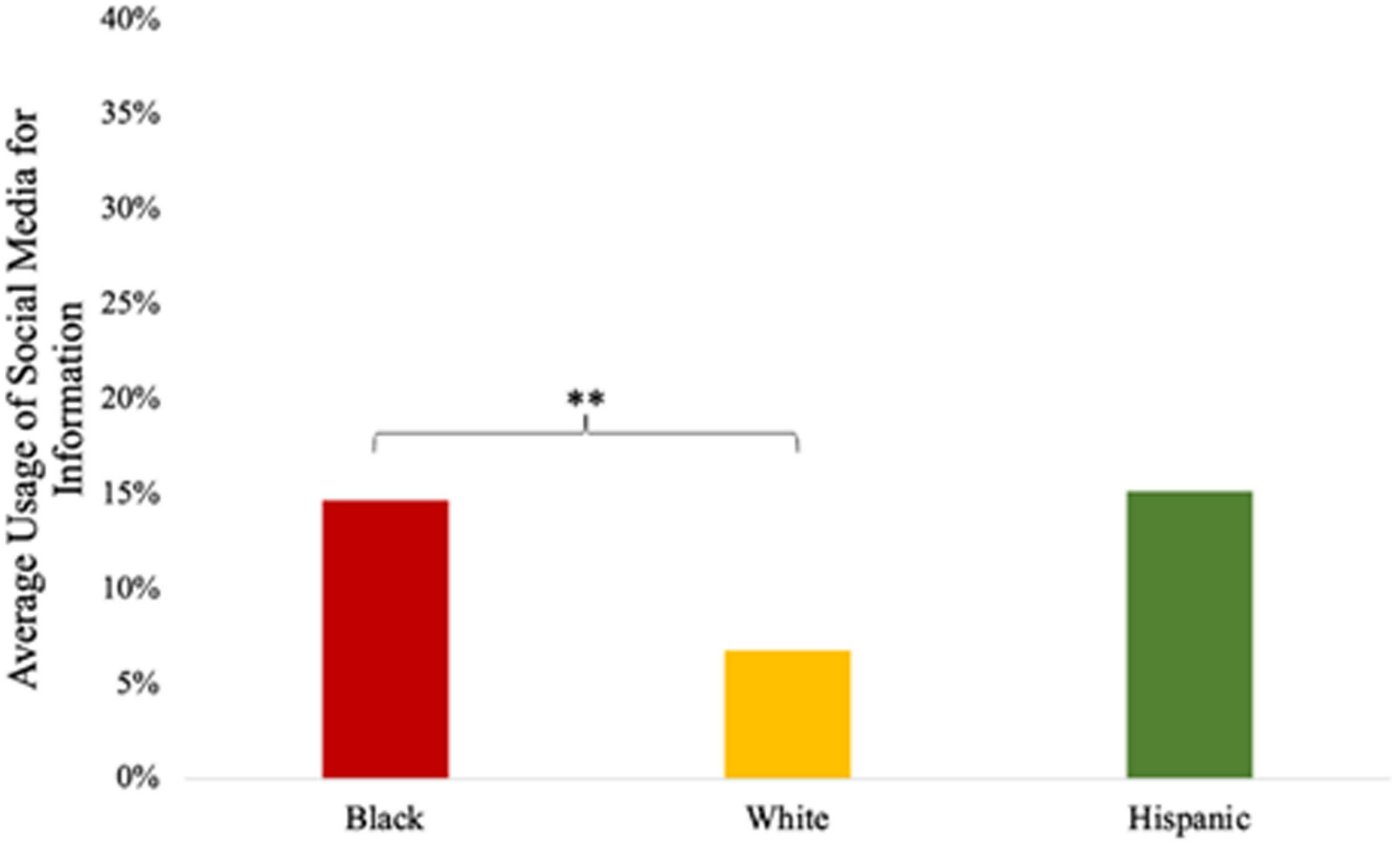
Social media as an information source for breast reconstruction. Black patients were significantly more likely to use social media as a mechanism to gather information about BR surgery compared to White patients (**p < 0.01). Total respondents = 378 (White = 250, Black = 99, Hispanic = 29). Asian and AI/AN groups were not included due to small sample sizes, which limited statistical power. Asterisks indicate statistical significance: **p < 0.01.

In terms of social media engagement, when asked which factors contributed to their decision to undergo BR, Black and Hispanic patients selected social media significantly more than white patients (*p* < 0.001 and *p* = 0.003, respectively). Concerning what information sources were consulted when gathering information about BR surgery, Black patients selected social media significantly more compared to white patients (*p* = 0.011) (Figures 3 and 4).

### 3.5 BREAST‐Q Subscale Results

Multivariate logistic regression analysis of the Breast‐Q satisfaction with breasts subscale showed that overall, white women were significantly less satisfied with their breasts compared to all other race groups (Effect size = −6.2 points, 95% CI: [−12.3, −0.1], *p* = 0.047)), after controlling for age, education, income, and type of BR. In terms of procedure type, multivariate analysis demonstrated a 22.8 [−25.9, −19,8] point lower BREAST‐Q satisfaction score in those receiving implant‐based versus autologous BR, while controlling for age, race, education, and income (*p* < 0.001).

## 4 Discussion

### 4.1 Racial Differences in Breast Cancer Treatment

Race was significantly associated with breast cancer treatment type, independent of stage. Black and Hispanic women were more likely to undergo aggressive surgery and less likely to receive breast‐conserving surgery (BCS) than white women, despite similar stages. The former highlights an ongoing disparity in clinical decision‐making [14, 15]. Pairwise analysis showed that Black women more often presented with early‐stage disease, yet still underwent higher rates of chemotherapy and mastectomy, despite comparable outcomes between BCS and mastectomy comparable in early‐stage cases [16, 17]. These findings raise concerns about systemic biases and inequities including the possibility that providers may unconsciously recommend more aggressive treatments for women of color [18]. However, due to the cross‐sectional design, we cannot distinguish provider influence from external factors such as social media.

Greater social media use among Black and Hispanic women adds complexity, as online information may shape preferences that defer from clinical guidance. Regardless of the underlying cause of these racial differences, it is also important to address the implications in terms of quality of life. aggressive treatments can negatively impact physical and psychological well‐being [19, 20]. In contrast, Asian women were significantly less likely to receive chemotherapy or radiation despite similar stages, raising concerns about potential under‐treatment and systemic barriers such as access, cultural factors, or informational gaps [21, 22, 23].

White women, despite higher BCS rates, reported lower breast satisfaction than non‐white women. Social media was associated with higher Breast‐Q scores, likely reflecting greater engagement and access to culturally revenant support and information [24].

### 4.2 Breast Reconstruction Complications and Shared Decision‐Making

Complications after breast reconstruction were associated with younger age, tobacco use, radiation, diabetes, heart disease, and autologous breast reconstruction. While autologous reconstruction is linked to higher satisfaction, it carries greater complication risk [25, 26]. Radiation contributes to delayed healing and capsular contracture [27, 28, 29]. Given these risks, reconstruction decisions should emphasize informed, patient‐centered counseling, including long‐term outcomes, revision needs, and individual trade‐offs between autologous and implant‐based approaches [30, 31, 33]. For patients undergoing radiation, timing and reconstructive strategy should be considered during the pre‐operative planned [29, 30, 31, 32, 33, 34].

### 4.3 Modifiable Risk Factors in Breast Reconstruction Surgery Outcomes

Tobacco use was the strongest independent risk factor for complications after adjustment (Table 5). Smoking cessation should be emphasized as stopping at least 4–6 weeks preoperatively reduces risk [34].

Black and Hispanic women have higher rates of comorbidities such as obesity, hypertension, and diabetes, contributing to poorer outcomes [35, 36, 37]. Our study similarly found higher rates of diabetes and hypertension among Black patients. Delayed reconstruction in diabetic patients has been associated with fewer complications, including infections and reoperations [37]. Considering our findings of a higher incidence of diabetes among Black patients, it is essential to implement strategies to Optimizing diabetes management is therefore critical, though barriers such as limited access and transportation may complicate care. Telehealth interventions have been shown to improve glycemic control among Black and Hispanic patients (HbA1c reduction −0.465 [CI: −0.648 to −0.282]) [38]. Incorporatingtelehealth in preoperative care may improve outcomes in these populations [39].

### 4.4 Reliance on Social Media for Information

Women of color were more likely to use social media for breast cancer‐information, consistent with prior findings of mistrust in healthcare and a lack of culturally tailored resources [40, 41]. However, motivations for social media use were not assessed.

While social media improves access, it may also spread misinformation that influences treatment decisions [42, 43]. Our study found that white women most commonly reported physician/medical personnel as their primary information source (77.7%), followed by medical websites (38.0%) and family/friends (36.4%), with only 13.2% reporting social media use. These differences may reflect variation in access, trust, and communication styles [44]. Understanding these patterns can inform culturally tailored strategies that leverage social media to deliver accurate information.

### 4.5 Intersecting Barriers and Opportunities for Change in Breast Cancer Care

These findings highlight the intersection of race, informational access, and treatment patterns. Although reasons for social media use remain unclear, differences in information‐seeking and treatment suggest underlying social and systemic influences. Patient decision aids may help address informational gaps and support shared decision‐making [45].

Lower rates of chemotherapy and radiation among Asian women further emphasize the need for culturally tailored interventions. While treatment differences should be interpreted cautiously due to survey limitations, social media and decision‐support tools offer opportunities to improve education, access, and equitable care.

### 4.6 Strengths and Limitations

This study's strengths include recruiting participants from diverse regions, healthcare systems, and socioeconomic backgrounds, enhancing sample heterogeneity. Web‐based methods via Kantar facilitated rapid inclusion of underrepresented groups and individuals outside traditional care settings, providing a broad view of patient experiences. The diversity of the sample enabled multivariable regression analyses, yielding meaningful insights into racial and ethnic differences in BC treatment and social media engagement, highlighting the relevance of the data captured

Limitations include potential bias toward participants with internet access and digital literacy, which may limit generalizability. We also lacked information on treatment sites and specific characteristics of medical centers. Timing of BREAST‐Q completion varied (6 months to over a year post‐surgery), which may affect patient‐reported satisfaction and quality of life. Future studies should consider standardizing survey timing or adjusting for time since surgery in analyses. Self‐reported data on cancer stage, treatment, and outcomes may introduce recall bias and limit interpretation of observed differences, such as chemotherapy or radiation use among Asian patients, since the survey did not capture whether these treatments were recommended or declined. Additionally, nuanced factors influencing surgical decisions, including shared decision‐making, may not be fully captured.

Despite these limitations, the study provides valuable insights into social media engagement and its role in breast cancer decision‐making among a diverse group of survivors. While treatment‐related findings should be interpreted cautiously, the results highlight the potential for leveraging social media to inform patient‐centered care, support decision‐making, and guide future research in surgical education and cancer information dissemination.

## 5 Conclusions

The combination of aggressive treatment patterns and frequent social media use among women of color reveals a critical opportunity to address persistent inequities in breast cancer care. Enhancing patient understanding of risks and strategically leveraging social media can help create more equitable, patient‐centered surgical experiences. Future research should focus on integrating evidence‐based decision aids with social media platforms to bridge informational gaps in breast reconstruction. This approach is especially promising for survivors of color, who often rely on social media for guidance. By uniting the credibility of decision aids with the reach and relatability of social media, we can deliver culturally relevant tools that inform, empower, and promote equity in care.

## Conflicts of Interest

R.D.G. serves as a consultant and member of the Medical Board of Directors for MTF. The other authors declare no conflicts of interest.

## Synopsis

This study examines racial disparities in breast cancer treatment, breast reconstruction outcomes, and information access among a diverse cohort of 413 breast cancer survivors, highlighting how women of color disproportionately undergo more aggressive treatments and rely more heavily on social media during surgical decision‐making. The findings reveal differences in chemotherapy, mastectomy, and reconstruction experiences across racial groups, while also demonstrating that social media plays a powerful and increasingly influential role in shaping patient perceptions, support systems, and satisfaction with care. By uncovering inequities in both clinical treatment patterns and access to culturally relevant information, this research underscores the urgent need for patient‐centered, evidence‐based tools and inclusive digital resources to improve equity in both breast cancer care and reconstruction outcomes.

## Supporting information

Supporting File 1

Supporting File 2

Supporting File 3

Supporting File 4

## Data Availability

The data that support the findings of this study are available from the corresponding authors, T.W. and R.D.G., upon reasonable request.
