# Supplementary material for: Racial Differences in Breast Cancer Treatment and Information Access
Source: J Surg Oncol. 2026 Jul 5;134(3):383–93. doi: 10.1002/jso.70304 (PMC13532962; doi:10.1002/jso.70304)
Supplement: Supplementary file 1 [file JSO-134-383-s001.pdf]

BREAST-Q™ - RECONSTRUCTION MODULE (POSTOPERATIVE) VERSION 2.0: SATISFACTION WITH BREASTS

If you have had a mastectomy and reconstruction of both breasts, answer these questions thinking of the breast you are least satisfied with. With your breasts in mind, in the past week, how satisfied or dissatisfied have you been with:

	Very Dissatisfied	Somewhat Dissatisfied	Somewhat Satisfied	Very Satisfied
a. How you look in the mirror clothed?	1	2	3	4
b. The shape of your reconstructed breast(s) when you are wearing a bra?	1	2	3	4
c. How normal you feel in your clothes?	1	2	3	4
d. The size of your reconstructed breast(s)?	1	2	3	4
e. Being able to wear clothing that is more fitted?	1	2	3	4
f. How your breasts are lined up in relation to each other?	1	2	3	4
g. How comfortably your bras fit?	1	2	3	4
h. The softness of your reconstructed breast(s)?	1	2	3	4
i. How equal in size your breasts are to each other?	1	2	3	4
j. How natural your reconstructed breast(s) looks?	1	2	3	4
k. How naturally your reconstructed breast(s) sits/hangs?	1	2	3	4
l. How your reconstructed breast(s) feels to touch?	1	2	3	4
m. How much your reconstructed breast(s) feels like a natural part of your body?	1	2	3	4
n. How closely matched (similar) your breasts are to each other?	1	2	3	4
o. How you look in the mirror unclothed?	1	2	3	4

BREAST-Q VERSION 2.0 © 2017 Memorial Sloan Kettering Cancer Center and The University of British Columbia. All rights reserved.

Note to Investigators: This scale can be used independently of the other scales.
