# Supplementary material for: Racial Differences in Breast Cancer Treatment and Information Access
Source: J Surg Oncol. 2026 Jul 5;134(3):383–93. doi: 10.1002/jso.70304 (PMC13532962; doi:10.1002/jso.70304)
Supplement: Supplementary file 2 [file JSO-134-383-s002.pdf]

NOTE TO LICENSED USERS

Each scale can be used independently of the other scales (i.e., you don't have to use them all). For each scale that you use, the patients or research participants DO NOT NEED TO SEE:

- title of each scale
- notes at the bottom of the scale
- scoring table for the scale

We are able to provide you with a Word version of this booklet if needed. Send an email to: qportfolioteam@gmail.com

Patients or research participants only need to see the instructions, items, response options and the copyright notice at the bottom of the scale. Here's an example:

With your breast area in mind, in the past week, how satisfied or dissatisfied have you been with:

	Very Dissatisfied	Somewhat Dissatisfied	Somewhat Satisfied	Very Satisfied
a. How you look in the mirror clothed?	1	2	3	4
b. How comfortably your bras fit?	1	2	3	4
c. Being able to wear clothing that is more fitted?	1	2	3	4
d. How you look in the mirror unclothed?	1	2	3	4

BREAST-Q VERSION 2.0 © 2017 Memorial Sloan Kettering Cancer Center and The University of British Columbia. All rights reserved.

AS A REMINDER: In the license you signed, you agreed to the following:

1. You will not change this questionnaire in any way
2. You will not translate this questionnaire without permission
3. You will not give this questionnaire to an unlicensed user
4. You will not reproduce this questionnaire in publications or other materials
