# Supplementary material for: Racial Differences in Breast Cancer Treatment and Information Access
Source: J Surg Oncol. 2026 Jul 5;134(3):383–93. doi: 10.1002/jso.70304 (PMC13532962; doi:10.1002/jso.70304)
Supplement: Supplementary file 3 [file JSO-134-383-s003.pdf]

Q-PORTFOLIO LICENSE AGREEMENT

IMPORTANT – PLEASE READ CAREFULLY

This Q-Portfolio License Agreement (this "Agreement") is a legally binding agreement between you and all persons you represent, including any natural persons and any type of incorporated or unincorporated entity ("You" or the "Licensee," interchangeably) and Memorial Sloan-Kettering Cancer Center ("MSK") for the Q-Portfolio questionnaire(s) you've requested access to such as BODY-Q, BREAST-Q, or FACE-Q (individually or collectively the "Questionnaire"), as well as all accompanying documentation and translations (together with the Questionnaire, the "Licensed Work"). Licensee and MSK may be referred to individually as "Party" and collectively as the "Parties."

IF YOU AGREE TO BE BOUND BY THE TERMS OF THIS LICENSE, CLICK THE 'YES' BOX IN THE REDCAP LICENSING FORM. IF YOU DO NOT AGREE TO THE TERMS OF THIS LICENSE, YOU ARE NOT AUTHORIZED TO USE THE LICENSED WORK.

By clicking on "Yes" in the REDCap licensing form, You agree, and will ensure all persons You represent agree, to be bound by the terms of this Agreement and You represent and warrant that You have the legal authority to enter into this Agreement on behalf of You and all persons You represent.

BACKGROUND

i. MSK is responsible for licensing of the Licensed Work.

ii. Licensee desires to acquire a license to use the Licensed Work for the following purpose (the "Permitted Purpose"): The use of the Licensed Work by Licensee's students/researchers, including use of the Licensed Work at the locations submitted by Licensee, as is reasonable or necessary for the performance of one academic, non-commercial study submitted by Licensee (the "Academic Study"), including uses by Licensee and its subcontractors, representatives, and agents for the analysis and use of the data in connection with the Academic Study.

AGREEMENT

In consideration of the benefits that the Parties will receive from this Agreement and for other good and valuable consideration (the receipt and sufficiency of which is hereby acknowledged by each of the Parties), the Parties agree as follows:

1. GRANT OF LICENSE

Subject to the terms and conditions of this Agreement and effective as of the date when You first click your acceptance to this Agreement (the "Effective Date"), MSK hereby grants to Licensee and Licensee hereby accepts a non-exclusive, non-transferrable, non-assignable, royalty-free license to use the Licensed Work for the Permitted Purpose. If any details of the Permitted Purpose change, Licensee must notify MSK at qotdtrm@mskcc.org and email the Q-Portfolio team at qportfolioteam@gmail.com.

2. PROHIBITED USES

Licensee shall not make any other use of the Licensed Work except as expressly permitted in this Agreement or in a separate license agreement with MSK. In particular, but without limitation, Licensee

shall not:

i. Modify, adapt, or create any derivative work of the Licensed Work, except as expressly set forth in Section 7 (“Translations”) of this Agreement;
ii. Sell, sublicense, rent, loan, transfer, or otherwise distribute the Licensed Work to any other person other than as required, and expressly permitted under this Agreement, in connection with the Permitted Purpose;
iii. Publicly perform or display the Licensed Work or any part of the Licensed Work, except for the administration of the Licensed Work in connection with the Permitted Purpose;
iv. Remove or obscure any copyright, trademark, or other proprietary notice contained in or associated with the Licensed Work; or
v. Perform any “Commercial Use,” defined as use of the Licensed Work, resulting data, or any portion thereof, directly or indirectly, (i) by or on behalf of a healthcare payor; (ii) for validation of a commercial product or regulatory approval of a product candidate; (iii) for marketing purposes for commercial products, including without limitation use for a clinical study on any product or product candidate sponsored or supported by or otherwise involving a for-profit entity other than a private hospital; or (iv) for marketing activities promoting Licensee’s services (e.g., treatments, diagnoses, or surgeries).

3. DELIVERY OF THE LICENSED WORK

After the Licensee has executed this Agreement, the Licensee will be able to download the Licensed Work from the website.

4. ACKNOWLEDGMENT OF INTELLECTUAL PROPERTY RIGHTS

Licensee acknowledges that the Licensed Work is protected by copyright, trademark, and other intellectual property rights. Licensee acknowledges that MSK administers all rights in the Licensed Work and that Licensee has no right to use the names “Memorial Sloan Kettering Cancer Center,” “FACE-Q,” “BREAST-Q,” or “BODY-Q,” except as is incidental to the Permitted Purpose. For current copyright, trademark, and other notices, Licensee should consult the Q Portfolio website, https://qportfolio.org/.

5. PUBLICATION

Licensee may publish research results based on data obtained using the Licensed Work, provided that Licensee does not publish any part of the Licensed Work and that all publications include the relevant copyright, trademark, or other notices as listed on the Q Portfolio website and the following statement: “The content and design of the questionnaire are protected by U.S. and international intellectual property laws, and use was made under a license from Memorial Sloan Kettering Cancer Center, New York, NY, USA. For further information on a permitted use or a license to use any content, contact MSK at qotdtrm@mskcc.org or qportfolioteam@gmail.com.”

6. CONFIDENTIALITY

6.1 Licensee acknowledges that the terms in this Agreement are relevant to this Agreement only and will not be considered as a template or precedent for any future agreements. Except as required by law, neither Party will disclose the terms of this Agreement to any third party without the prior written consent of the other Party. Neither Party will use the name of the other Party in any publicity, advertising, or announcement without the prior written approval of the other Party.

6.2 MSK will keep confidential any information submitted to it by the Licensee under this Agreement,

and will use due diligence to prevent disclosure of such information, except to its employees as necessary for the conduct of its obligations under this Agreement who are bound by similar written obligations of confidentiality for a period of five (5) years after the execution of this Agreement.

7. TRANSLATIONS

7.1 Licensee may use the Licensed Work in any of the languages available from MSK which have been linguistically validated and for which a Certificate of Translation is available.

7.2 MSK has in its possession translations of the Licensed Work which have been linguistically validated in different languages, either professionally or by local academics. MSK may, in its discretion, provide these translations to Licensee for the Permitted Purpose, provided that Licensee acknowledges and agrees in writing that some translations were not certified by a professional translation firm and may have errors that could affect interpretation and use.

7.3 Licensee may translate the Licensed Work, including into any languages which are not presently available through this website, but only in accordance with the Translation Protocol attached hereto as Schedule A. If Licensee wishes to translate the Licensed Work, Licensee shall email the Q-Portfolio team at qportfolioteam@gmail.com prior to entering into this Agreement.

7.4 If the Q-Portfolio team grants Licensee the right to translate the Licensed Work or any portion thereof, Licensee agrees to provide a copy of the translation as soon as possible to the Q-Portfolio team at qportfolioteam@gmail.com. Such translation will be considered part of the Licensed Work under this Agreement and shall be subject to all the terms herein.

8. DISCLAIMER OF WARRANTIES

EXCEPT FOR THE REPRESENTATIONS AND WARRANTIES EXPRESSLY SET FORTH HEREIN, THE LICENSED WORK IS PROVIDED BY MSK “AS IS,” AND MSK DISCLAIMS ALL OTHER WARRANTIES, EXPRESS OR IMPLIED, INCLUDING, WITHOUT LIMITATION, ANY IMPLIED WARRANTIES OF MERCHANTABILITY OR FITNESS FOR A PARTICULAR PURPOSE WITH RESPECT TO THE LICENSED WORK LICENSED HEREUNDER. MSK DOES NOT WARRANT THE VALIDITY OF ANY INTELLECTUAL PROPERTY RIGHTS IN OR TO THE LICENSED WORK AND MAKES NO REPRESENTATION WHATSOEVER THAT THE LICENSED WORK MAY BE EXPLOITED BY LICENSEE WITHOUT INFRINGING OTHER INTELLECTUAL PROPERTY RIGHTS.

THE LIABILITY OF MSK, ITS AGENTS, OR ITS EMPLOYEES, WITH RESPECT TO ANY AND ALL SUITS, ACTIONS, LEGAL PROCEEDINGS, CLAIMS, DEMANDS, DAMAGES, COSTS, AND EXPENSES ARISING OUT OF THE PERFORMANCE OR NON PERFORMANCE OF ANY OBLIGATION UNDER THIS AGREEMENT, WHETHER BASED ON CONTRACT, WARRANTY, TORT (INCLUDING WITHOUT LIMITATION NEGLIGENCE), STRICT LIABILITY, STATUTE, OR OTHERWISE WILL BE LIMITED TO DIRECT, ACTUAL DAMAGES INCURRED AS A RESULT OF MSK’S FAILURE TO PERFORM ITS OBLIGATIONS AS REQUIRED BY THIS AGREEMENT AND EVEN IF ADVISED OF THE POSSIBILITY OF SUCH DAMAGES, MSK WILL IN NO EVENT BE LIABLE FOR ANY LOST PROFITS, LOST BUSINESS OPPORTUNITY OR INDIRECT, SPECIAL, INCIDENTAL, CONSEQUENTIAL, OR PUNITIVE DAMAGES OF ANY KIND.

9. INDEMNITY AND LIMITATION OF LIABILITY

9.1 In no event will MSK be liable for any use by Licensee of the Licensed Work, including any portion, translation, or derivative work thereof, whether authorized or unauthorized, or for any suits, actions,

legal proceedings, costs, claims, demands, damages, expenses (including, but not limited to, reasonable legal, expert, and consultant fees), or liability of any kind whatsoever arising directly or indirectly from such use of the Licensed Work or the performance or non-performance of any obligation under this Agreement, whether based on contract, warranty, tort (including without limitation negligence), strict liability, statute, or otherwise (collectively "Claims").

9.2 Licensee will indemnify, defend, and hold harmless MSK and its respective trustees, directors, officers, medical and professional staff, employees, students, agents, and affiliates and their respective successors, heirs, and assigns from and against any Claims. Where Licensee is prohibited or restricted by applicable law to indemnify or hold harmless MSK, Licensee shall be responsible to the full extent permitted by applicable law for any Claims.

10. INSURANCE

Licensee shall maintain liability insurance sufficient to protect Licensee and MSK in regard to the events covered above by Section 9 ("Indemnity and Limitation of Liability") of this Agreement.

11. TERM and TERMINATION

11.1 This Agreement commences on the Effective Date and terminates upon the earlier of the following: (a) twelve (12) months after the conclusion of the Academic Study; or (b) the completion date of the Permitted Purpose.

11.2 Either Party may terminate this Agreement for a material breach of this Agreement, provided that the breaching Party fails to cure such material breach within sixty (60) days after receipt of written notice specifying such material breach.

11.3 In the event that a Party becomes insolvent, makes an assignment for the benefit of creditors, files for bankruptcy, or ceases or threatens to cease to carry on the whole or any relevant part of its business or trade (hereafter an "Insolvent Party"), the other Party may terminate this Agreement effective upon delivery of a written notice to the Insolvent Party.

11.4 Upon termination or expiration of this Agreement for any reason, nothing in this Agreement may be construed to release either Party from any obligation (such as any license fees) that accrued prior to, or that is expressly indicated to survive, the effective date of the termination or expiration, as applicable. Notwithstanding the termination or expiration of this Agreement, the rights and obligations in Sections 4, 5, 6, 8, 9, and 10 of this Agreement will survive and continue to bind the Parties and each of their successors and assigns.

12. GENERAL PROVISIONS

12.1 Assignment and Subcontracting
Neither Party shall sublicense or assign this Agreement to any third party without receiving prior written permission from the other Party, provided that a Party shall be entitled to assign its rights and obligations hereunder in connection with the sale of all, or substantially all, of its business related to the Permitted Purpose or its obligations under this Agreement.

12.2 Entire Agreement
This Agreement contains the entire agreement between the Parties with regard to the Licensed Work and supersedes all prior oral or written communications or understandings between the Parties. No

amendments to this Agreement shall be valid or enforceable unless in writing and signed by all Parties.

12.3 Severability
If any provision of this Agreement is wholly or partially invalid, illegal or unenforceable for any reason, all other provisions will continue in full force and effect.

12.4 Binding Effect
This Agreement enures to the benefit of and is binding upon the Parties and their respective successors, executors, administrators or other legal representatives and permitted assigns.

12.5 Notices
All notices or other communications to be given under this Agreement by one Party to the other shall be in writing and shall be delivered in one of the following formats: electronically, by prepaid courier, by registered prepaid mail, or by hand. Such notices will be deemed received upon the earlier of confirmation of such receipt or seven (7) days. Notices to MSK must be addressed to :

Office of Technology Development
Memorial Sloan Kettering Cancer Center
1275 York Ave, New York, NY 10065
qotdtrm@mskcc.org

12.6 No Waiver
Failure of a Party to enforce its rights on one occasion will not result in a waiver of those rights on any other occasion.

12.7 Applicable Law and Jurisdiction
This Agreement, and all claims arising out of or relating to this Agreement, will be construed, governed, interpreted, and applied in accordance with the laws of the State of New York, without regard to any conflicts of law principles. The state and federal courts located in New York County, New York, will have exclusive jurisdiction of any claims or actions between or among the Parties arising out of or relating to this Agreement or the relationship between the Parties, and each Party consents to venue and personal jurisdiction of those courts for the purpose of resolving any such disputes..

12.8 Copy of Agreement
Licensee should print out or download a copy of this Agreement and retain it for their records.

12.9 Language
Licensee consents to the use of the English language or a Translation in this Agreement.

SCHEDULE A

TRANSLATION PROTOCOL

1. Licensee shall obtain, review and abide by the Q-Portfolio document entitled: *A Guide for Translation and Cultural Adaptation of the Q-Portfolio Questionnaires*. If Licensee or the translation company has any questions regarding the translation protocol, Licensee and/or the translation company will arrange and participate in a conference call with a member of the Q-Portfolio team before beginning any translation work;

2. Licensee is responsible for hiring the services of translators or a translation company of their choosing and introducing the translation company to the Q-Portfolio team.

3. Licensee is responsible for all fees related to the translation process and acknowledges that the universities that own the Q-Portfolio Questionnaire retain sole rights and ownership to each translation.

4. Licensee acknowledges they may be required to enter into a separate consulting contract with the creator for his/her review and approval of translations, which is separate from this Agreement. The Licensee will liase with a member of the Q-Portfolio team to arrange for the consulting work to be completed.

5. Licensee shall provide the Q-Portfolio team with a planned timeline for preparation of all translations before beginning any translation work.

6. Licensee will ensure the translation company's process follows the ISPOR guidelines as described in *A Guide for Translation and Cultural Adaptation of the Q-Portfolio Questionnaires*, which includes the following standard steps:
    i. Conceptual analysis of source document(s) provided by McMaster University.
    ii. Two, independent forward translations into target language.
    iii. Reconciliation and harmonization of the 2 forward translations; Preparation of version 1 of the target language.
    iv. One back-translation (target language → source language).
    v. Comparison of back translation to original source language version. Harmonization based on developers feedback. Preparation of version 2 of target language.
    vi. Cognitive debriefing interviews with at least 5 participants.
    vii. Review of cognitive debriefing interview results. Preparation of the final version in the target language. Finalization and proofreading.
    viii. The Q-Portfolio team is provided with all documentation related to the translation development, including back translation reviews.
    ix. The Q-Portfolio team will format the final translation(s) in Word and PDF formats, along with a copy of the translation certificate where applicable.

7. The validation of any translation of a Q-Portfolio questionnaire shall be at the sole discretion of McMaster University.

8. The ownership of and copyright to any translation will be the sole property of the university that owns the Q-Portfolio Questionnaire; and

9. Each authorized translation must include the copyright notice for the Q-Portfolio Questionnaire in English and the target language.
