# Supplementary material for: Racial Differences in Breast Cancer Treatment and Information Access
Source: J Surg Oncol. 2026 Jul 5;134(3):383–93. doi: 10.1002/jso.70304 (PMC13532962; doi:10.1002/jso.70304)
Supplement: Supplementary file 4 [file JSO-134-383-s004.pdf]

BRAVE Patient Access Survey
(Final Version)

Objectives:
● Understand to what extent women currently have good access to breast reconstruction services after having undergone breast surgery for the treatment of breast cancer.
● Characterize the gaps in care that may exist, including the availability of plastic surgeons and the role of breast surgeons.
● Evaluate the role of race/ethnicity, socioeconomic status, and social media in breast reconstruction trends and outcomes.

Kantar Instructions/Participant Demographics:

Approximately 400 patients from the racial/ethnic groups are listed below:
White/Caucasian
MIN. 0 – MAX 250
Black or African American
MIN. 70 – MAX. 320
Hispanic non-White or Latina
MIN. 50 – MAX. 300
American Indian or Alaska Native/ Native Hawaiian or Other Pacific Islander
MIN. 10 – MAX. 260
Asian
MIN. 20 – MAX. 270

SCREENER
Thank you for agreeing to take this survey. Your responses will be strictly anonymous and will be used to better inform women in the future about their options during their breast cancer journey.

1. Please select your appropriate age range:
a) Less than 18 years of age **SCREEN OUT**

b) 18-24

c) 25-34

d) 35-44

e) 45-54

f) 55-64

g) 65 or over

2. What is your sex?

a) Female

b) Male **SCREEN OUT**

c) Transgender **SCREEN OUT**

d) Other **SCREEN OUT**

3. Please select your race/ethnicity (Please select **only one**)

[RANDOMIZE]

a) White/Caucasian

b) Black or African American

c) Hispanic non-White or Latina

d) American Indian or Alaska Native/ Native Hawaiian or Other Pacific Islanders e) Asian

f) Unknown **[ANCHOR] SCREEN OUT**

g) Prefer not to answer **[ANCHOR] SCREEN OUT**

4. Are you a breast cancer survivor?

a) Yes

b) No **SCREEN OUT**

5. Were you first diagnosed with breast cancer within the last 15 years?

a) Yes

b) No **SCREEN OUT**

5a. Have you lived in the US continually since your breast cancer diagnosis?

a) Yes

b) No **SCREEN OUT**

6. Which of the following procedures did you receive? (Please select **only one**)

a) Mastectomy (unilateral)

b) Mastectomy (bilateral)

c) Breast Conserving Surgery (Lumpectomy) or Oncoplastic Breast Surgery **SCREEN OUT**

d) None of these **SCREEN OUT**

6b. What was your approximate age at the time of your breast cancer diagnosis? a) Less than 35
b) 35-44
c) 45-54
d) 55-64
e) 65 and over **[SCREEN OUT]**

7. What factors contributed to your decision to undergo the **[INSERT Q6]**? (Please select **all** that apply)
[RANDOMIZE]
a) Out of Pocket Cost
b) Insurance Coverage/Co-pay cost
c) Previous Medical Debt
d) Breast Symmetry
e) Recommendation of a Healthcare worker
f) Recommendation of Family or Friend(s)
i) The Surgeon shared my racial/ethnic background
g) Social Media
h) Other (SPECIFY) **(OE) [ANCHOR]**

END SCREENER.

[MAIN SURVEY]

INTRO [SHOW ON ITS OWN SCREEN]

I'm Dr. Williams, a ten-year breast cancer survivor. I currently work as the Research Fellow for the Division of Plastic Surgery at Northwestern Feinberg School of Medicine. My focus is health disparities and outcomes research in breast cancer survivors. The purpose of this survey is to gather information about your breast cancer journey. Your participation will give us information on how to optimize access to quality care for breast cancer survivors. ***Your voice matters!*** *Thank you.*

8. What stage was your breast cancer at the time of your diagnosis?

a) Stage 0 or Ductal carcinoma in situ (DCIS)

b) Stage 1

c) Stage 2

d) Stage 3

e) Stage 4

f) Unsure

9. Were you diagnosed with one of the following types of breast cancer?

[RANDOMIZE]

a) HER 2- positive

b) Triple-Negative

c) None of the above **[ANCHOR]**

d) Unsure

10. Did you undergo chemotherapy as a part of your breast cancer treatment?

a) Yes

b) No

c) Unsure

11. Did you undergo radiation therapy as a part of your breast cancer treatment?

a) Yes

b) No

c) Unsure

12. Please indicate your current…

a) Weight:

____ lbs. **[RANGE 40 – 700]**

b) Height:

____ ft. **[RANGE 3 – 7]**

____ in. **[RANGE 0 – 11]**

c) Prefer not to answer **[EXCLUSIVE]**

[CALCULATE BMI IF BOTH HEIGHT AND WEIGHT ARE ANSWERED]

13. Have you been diagnosed with any of the following disease conditions? (Please select **all** that apply)

[RANDOMIZE]

a) Heart disease

b) High blood pressure/Hypertension

c) Diabetes

d) Renal/Kidney disease

e) High cholesterol/hyperlipidemia

f) None of the above **[ANCHOR, EXCLUSIVE]**

14. Do you smoke tobacco products?

a) Yes

b) No

c) Prefer not to answer

15. Did you have breast reconstruction (restoration) surgery after your cancer surgery to improve the appearance of your breasts /chest?

a) Yes

b) No

[ASK Q16 – Q23 IF Q15 = NO; SHOW Q16– Q21 ON SAME SCREEN]

16. Was **your age** a factor in your decision not to have breast reconstruction surgery?

a) Yes

b) No

c) Unsure

17. Was **lack of information** about breast reconstruction and its potential benefits a factor in your decision not to have breast reconstruction surgery?

a) Yes

b) No

c) Unsure

18. Was **lack of social support** (e.g., family or friends) a factor in your decision to not to have breast reconstruction surgery?

a) Yes

b) No

c) Unsure

19. Please share the reasons (outside of those listed above) why you chose not to undergo breast reconstruction surgery? **(OPTIONAL; OE)**

20. Were you aware of the federal law that provides protections to patients who choose to have breast reconstruction in connection with a mastectomy (The Women's Health and Cancer Rights Act of 1998-WHCRA)?

At the time of your breast cancer procedure(s), were you informed of the federal law that provides protections to patients who choose to have breast reconstruction in connection with a mastectomy (The Women's Health and Cancer Rights Act of 1998 (WHCRA)?

a) Yes

b) No

21. Thank you, breast cancer survivor, for your time in completing our BRAVE survey. We would like to know if there is anything that you would like to share about your experience. **(OE)**

[END OF SURVEY IF Q17 = NO]

[ASK REMAINDER OF SURVEY IF Q15 = YES]

[SHOW INTRO TEXT ON OWN SCREEN]

For the remainder of the survey, we would like to discuss your breast reconstruction surgery specifically.

22. Approximately how many years has it been since your breast reconstruction surgery? **[NUMERIC ENTRY, RANGE 0 –15]**

23. Did you have reconstruction surgery on one or both breasts?

a) One breast

b) Both breasts

c) Unsure

24. What type of breast reconstruction surgery did you have? (Please select **only one**)

[RANDOMIZE]

a) Implant-based reconstruction alone: Inserting an implant that's filled with saline (salt water) or silicone gel

b) Autologous or "flap" reconstruction: Using tissue transplanted from another part of your body such as your belly, thigh, or back

c) I am not sure **[ANCHOR]**

d) Other (SPECIFY) **(OE) [ANCHOR]**

25. Did you experience any immediate complications after your breast reconstruction surgery?

a) Yes

b) No

[ASK Q26 IF Q25 = YES]

26. Which complication(s) did you have?

(Please select **all** that apply)

[RANDOMIZE]

a) Seroma (a fluid collection at the site of your surgery)

b) Hematoma (a collection of blood at the site of your surgery)

c) Infection

d) Wound dehiscence (opening of your surgical incisions)

e) Flap compromise or loss (failure of your breast flap)

f) Capsular Contracture (tightening/hardening of the tissue around your breast implant(s)) Other (please describe): ____________ **(OE) [ANCHOR]**

27. What kind of surgeon performed your breast reconstruction surgery?

[RANDOMIZE]

a) A plastic surgeon

b) A breast surgeon (not a plastic surgeon)

c) A general surgeon (not a plastic surgeon)

d) I am not sure **[ANCHOR]**

28. Was your breast reconstruction surgery performed by a surgeon of your choice or a surgeon that you were assigned to?

[RANDOMIZE]

a) Surgeon of my choice

b) Surgeon I was assigned to (not my choice)

29. Which of the below were factors in the decision regarding what surgeon performed your reconstruction? (Please select **all** that apply)

[RANDOMIZE]

a) Out of Pocket Cost

b) Insurance Coverage/Co-pay cost

c) Previous Medical Debt

d) Breast Symmetry

e) Recommendation of a Healthcare worker

f) Recommendation of Family or Friend(s)

g) The Surgeon shared my racial/ethnic background

h) Social Media

j) Other (please explain): **(OE) [ANCHOR]**

30. What information sources did you consult for help regarding your breast reconstruction decisions? (Please select **all** that apply)

[RANDOMIZE]

a) My employer's human resources (HR) Department

b) Physician, Nurse or, Other medical personnel

c) Family or Friend(s)

d) Medical website

e) Social Media

f) Non-profit organization / website

g) Other (please explain): **(OE) [ANCHOR]**

(ASK Q31 IF 'F' FOR QUESTION 30)

31. What specific non-profit organization / website did you consult for help regarding your reconstruction decisions? **(OE)**

32. At the time of your cancer surgery, do you feel that you were properly informed about your options regarding breast reconstruction?

a) Yes

b) No

33. What options were you made aware of regarding breast reconstruction?

(Please select **all** that apply)

[RANDOMIZE]

a) Your right to breast symmetry (options regarding reconstructive surgery on one or both breasts)

b) Your insurance coverage and related costs for the unaffected breast

c) Nipple/areola tattooing

d) None of the above **[ANCHOR, EXCLUSIVE]**

34. At the time of your procedure(s), were you informed of the federal law that provides protections to patients who choose to have breast reconstruction in connection with a mastectomy (The Women's Health and Cancer Rights Act of 1998 (WHCRA)?

a) Yes

b) No
